# Leaf structure and seed histochemistry analyses provided structural insights into the improved yield and quality of tree peony seed under light shading conditions

**DOI:** 10.1038/s41598-020-61366-8

**Published:** 2020-03-09

**Authors:** Chenjing Han, Mei Yu, Qi Wang, Lei Wang, Haiping Yang, Yongjun Zhao, Hezhong Dong

**Affiliations:** 1 Cotton Research Center, Shandong Academy of Agricultural Sciences, Jinan, 250100 Shandong P. R. China; 2 Department of Food Science and Engineering, Shandong Agricultural and Engineering University, Jinan, 250100 Shandong P. R. China; 3 Shandong Forest Germplasm Resources Center, Jinan, 250102 Shandong P. R. China

**Keywords:** Light responses, Plant stress responses

## Abstract

In biology, structure is the basis of function. For plants, changes in their physiological and ecological functions are usually caused by structural changes. To understand how shading conditions change the plant structures, thereby providing structural insights into the improved yield and quality, oilseed tree peony were shaded with different densities of polyethylene nets from 28 days after pollination (DAP) until harvesting. The thickness of the leaf (LT), vein (VT), upper epidermis (UET), lower epidermis (LET), palisade tissue (PT), sponge tissue (ST), as well as the accumulation and distribution of starch, protein, and fat, were observed at 14-day intervals. The results showed that shading had a significant effect on the anatomical structure of the leaves. In the rapid growth period (before 70 DAP), the LT, ET, and VT under shading were significantly lower than under non-shading. During this period, the accumulation of starch and protein under shading was lower than that under non-shading. At the maturation period (99–112 DAP), the LT and PT under shading were higher than under non-shading, indicating that light shading delayed leaf senescence and increased photosynthetic capacity. Shading delayed the degradation of the integument cells and prolonged seed development and nutrient accumulation.

## Introduction

Tree peony (*Paeonia* section Moutan DC.) is indigenous to China, and its wild germplasms are mainly distributed in mountainous shrublands and secondary broad-leafed forests in the provinces of Henan, Gansu, and Shanxi^1^. In the transplantation process of oilseed tree peony from mountainous shrublands to the field, their leaves usually turn yellow, senescence prematurely, or even fall off due to the high light intensity and temperature, which seriously reduces the yield and quality of the tree peony seeds^2^. Shading is the preferred method for providing a suitable environment during the growth period since high light intensity and temperature are detrimental to ornamenta shrubs growth^3^. For example, when kiwi fruit plants were shaded, the canopy microclimate was significantly optimized, the temperature was lowered, and the humidity was increased, which effectively alleviated the physiological stress of the leaves and fruits and reduced the fallen fruit^4^. Under shading, *Trollius chinensis* Bunge grew relatively better and exhibited an extended viewing life^5^.

Light is a primary factor of the physical environment. Shading changes the microenvironment, which consequently alters leaf physiological characteristics^6^ as well as leaf structure^7–9^. Shading increases leaf area and reduces leaf thickness^7^ and reduces palisade tissue and stomatal density^8^. It also increases the intercellular space between the palisade tissue and spongy tissue, as well as the numbers of vascular bundles^5^. Shading has also been found to reduce the palisade tissue thickness, stomatal density, and the ratio of palisade tissue to sponge tissue^10^. Shading also affects substance distribution in plant tissues. The distribution of starch grains in basic tissues under 50% shading was greater than under non-shading^11^. Despite this information, the effects of shading on nutrient distribution in tree peony seeds remain unclear.

In our previous study, we found that light shading significantly increased the yield and quality of tree peony seed^2^. Subsequently, we explored the physiological mechanism whereby light shading increased seed yield and quality through the analysis of the seed development process, nutrient accumulation, and leaf physiological characteristics^6,12^. Structure is the basis of function and is responsible for the variation in plant physiology and ecology. In this work, our objectives were to determine: (i) the leaf anatomical structure; (ii) the effects of shading on leaf anatomical structure; and (iii) tree peony seed histochemistry. This research should provide a theoretical morphological and cytological reference for the cultivation of oilseed tree peony under light shading.

## Materials and Methods

### Shading treatment and experimental design

The shading experimental designs were the same as previously described^2,12^. The field experiment was conducted in an oilseed tree peony planting base at the Experimental Station of Shandong Cotton Research Center, Linqing (36 °61′ N, 115 °42′ E, 35 m a.s.l.), Shandong, China, in 2018. Two-year-old *Paeonia ostii* ‘Feng Dan’ seedlings transplanted in 2015 were selected for this study. The row length × row spacing was 30 cm × 50 cm. The average plant height before shading was 52.3 cm. The plot where the oilseed tree peony had consistently been grown was covered with three different densities of polyethylene net. The photosynthetic photon flux density (PPFD) through the three nets was reduced by 18.4%, 58.6%, and 82.1%, respectively, which measured with a Li-1400 Photometric System (Li-Cor, USA), corresponding to the three treatments of light, moderate, and severe shading^2,6,12^. The size of each polyethylene net was 8 m × 4 m. The treatments were arranged in a randomized complete block design with 3 replications. Shading was initiated at 28 days after pollination (DAP) and ended when seeds were ready for harvesting.

### Anatomical analyses

Leaves and seeds were collected starting at 42 DAP with 14-day intervals until seed harvest. The 3rd fully expanded young leaf on the main-stem from terminal were collected. To observe the anatomical structure of the mesophyll cells, a leaf sample was cut into 5-mm segments along the midrib and fixed in FAA (formalin: alcohol: glacial acetic acid was 90:5:5, V). The fixed material was then dehydrated using an ethanol series, cleared in xylene, embedded in paraffin wax, and then cut into 4–6-μm-thick sections using a rotary microtome (RM2016, Leica, Germany). The sections were stained with both safranine and fast green and fixed in neutral balata. They were subsequently observed and photographed with a microscope (Nikon Eclipse E100, Nikon digital sight DS-U3, Japan).

Tree peony seeds were fixed in FAA and 2.5% glutaraldehyde respectively. The sections fixed in FAA were stained with PAS (Periodic Acid Schiff) and naphthol sulfonic acid (Servicebio, Wuhan, China). The starch granules were stained to purplish red, the cell wall was also dyed purplish red, and the protein presented yellow. The seeds fixed in 2.5% glutaraldehyde were immersed in 1% citric acid and then embedded in paraffin sections. The sections were stained with Nile red (Servicebio, Wuhan, China) in the dark, and the lipids appeared orange-red or red under a fluorescent microscope.

### Data collection and analysis

The images were analyzed by the Image-Pro Plus 6.0 software (Media Cybernetics, USA). Sections with complete cells were selected and the following indices were measured: leaf thickness (LT), vein thickness (VT), upper epidermis thickness (UET), lower epidermis thickness (LET), palisade tissue thickness (PT), spongy tissue thickness (ST), and the ratio of palisade tissue thickness to spongy tissue thickness (P/S). All indexes were collected 30 data and subjected to analysis of variance (ANOVA) using the SPSS software (SPSS 19.0, Armonk, NY, USA).

## Results

### Leaf morphology of oilseed tree peony

**Figure 1:**
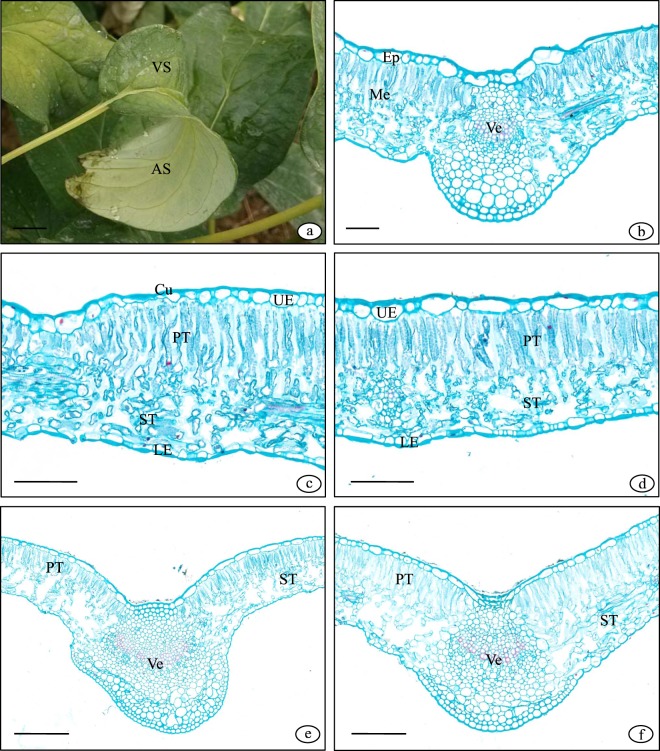
The leaf structure of oilseed tree peony. (**a**) Bifacial leaf with a ventral and abaxial surface; (**b**) Leaf cross-section; (**c**) Leaf structure under non-shading at 70 DAP; (**d**) Leaf structure under light shading at 70 DAP; (**e**) Leaf cross-section under non-shading at 112 DAP; (**f**) Leaf cross-section under light shading at 112 DAP. VS: ventral surface; AS: abaxial surface; UE: upper epidermis; PT: palisade tissue; ST: spongy tissue; Ep: epidermis; Me: mesophyll; Ve: vein; Cu: cuticle; LE: lower epidermis; MV: main vein; LV: lateral vein. Scale bars: (**a**), 1 cm; (**b)**, 100 μm; (**c,d**), 50μm; (**e,f**), 100 μm.

Oilseed tree peony possesses a bifacial leaf with a distinct ventral and abaxial surface (Fig. 1a). The structure of the leaf, from the outside to inside, includes an epidermis, mesophyll and veins (Fig. 1b). The epidermis is the outermost layer of the leaf and is divided into the upper epidermis and lower epidermis, each of which is composed of one living cell. This is the primary protective tissue (Fig. 1c). The cells are square and rectangular without chloroplasts and are arranged neatly with a thick cell wall and are evenly covered with cuticula. There are no stomata or obvious accessory structures such as trichomes or bristles in the upper epidermis. The stomata are distributed in the lower epidermis.

The mesophyll is located between the upper and lower epidermis and is connected to the upper epidermis, and the long axis of the mesophyll cell is perpendicular to the epidermis (Fig. 1c,d). The palisade tissue consists of dense columnar parenchyma containing many chloroplasts. The loose parenchyma cells between the palisade tissue and lower epidermis are spongy tissue. The spongy tissue cells are irregular in size and traits, with short arm-like projections and are interconnected (Fig. 1c,d). The well-developed interstitial space in the spongy tissue and sub-chamber form the leaf ventilation system. There are fewer chloroplasts in the spongy tissue cells.

The reticular veins are composed of main veins and lateral veins and are distributed in the mesophyll tissue (Fig. 1b). The main vein contains a vascular bundle, with the xylem located above (ventral surface) and the phloem located below (abaxial surface). There is a cambium between them, while its splitting ability was weak and activity time was short. Thus, the main vascular bundle is not well developed. Several layers of cells above the vascular bundle are differentiated into collenchyma tissue (Fig. 1b), and the outermost two layers of cells below are differentiated into sclerenchyma tissue, which enhances mechanical support.

### Effects of shading on epidermal cells

**Figure 2:**
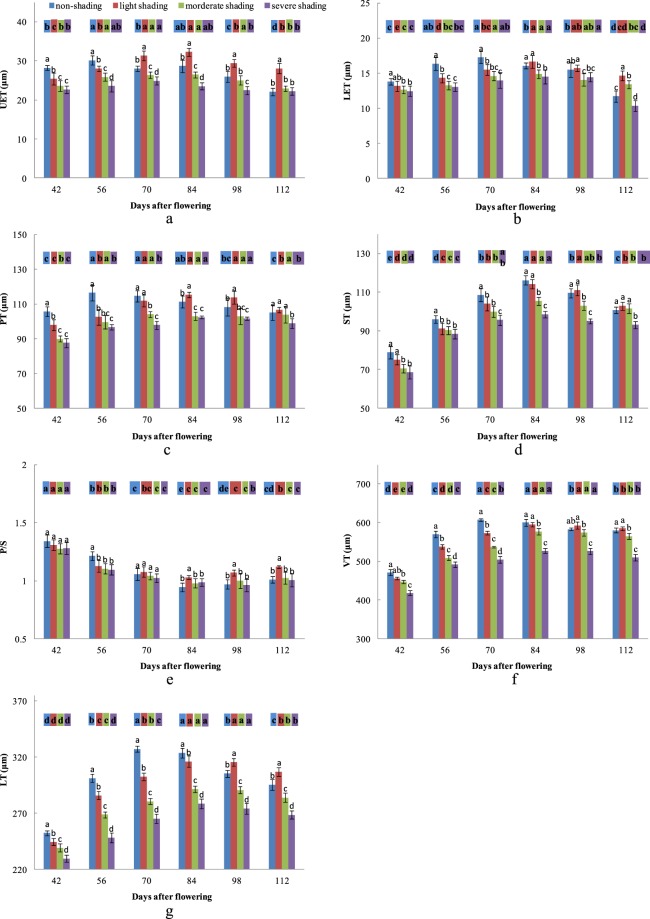
The effects of shading and collection time on UET, LET, PT, ST, P/S, VT, and LT. (**a**) Effects of shading and time on UET; (**b**) Effects of shading and time on LET; (**c**) Effects of shading and time on PT; (**d**) Effects of shading and time on ST; (**e**) Effects of shading and time on P/S; (**f**) Effects of shading and time on VT; (**g**) Effects of shading and time on LT.

Shading significantly affected UET (Fig. 2a). The UET under light, moderate, and severe shading was decreased by 10.0, 16.3, and 19.6% at 42 DAP, respectively, and 7.0, 14.2, and 21.8% at 56 DAP, respectively. However, light shading increased UET by 12.0% at 70 DAP, 12.4% at 84 DAP, 13.4% at 98 DAP, and 27.2% at 112 DAP. To investigate how different shading conditions affect the structure of the upper epidermis, we measured its thickness at different growth stages. The UET increased first and then decreased, and there were significant differences among the treatments. The UET reached the maximum at 56 DAP under non-shading, at 84 DAP under light and moderate shading, and at 70 DAP under severe shading.

Shading had significant effects on LET (Fig. 2b). The LET under light, moderate, and severe shading was decreased by 12.5, 19.0, and 20.2% at 56 DAP, and 10.1, 15.3, and 18.8% at 70 DAP. Nevertheless, light shading increased LET by 25.1% at 112 DAP. Similar to UET, the variation in LET with time was parabolic. The LET under non-shading reached the maximum at 70 DAP and then gradually decreased until 112 DAP. The LET under all shading treatment peaked at 84 DAP, while the minimum was observed at 42 DAP under light and moderate shading and at 112 DAP under severe shading.

### Effects of shading on the mesophyll cells

PT was significantly affected by shading (Fig. 2c). The PT under light, moderate, and severe shading was decreased by 7.3, 14.8, and 17.1% at 42 DAP, and 12.0, 14.4, and 17.0% at 56 DAP. On the contrary, light shading increased PT by 5.1% at 84 DAP and 7.3% at 98 DAP. The PT of all treatments increased first and then decreased, with significant differences observed between the treatments. The PT under non-shading reached the maximum at 56 DAP, and then gradually decreased to the minimum at 112 DAP. Under all shading, PT reached the maximum at 84 DAP and the minimum at 42 DAP.

Shading had significant effects on ST (Fig. 2d). The ST under light, moderate, and severe shading decreased by 5.0, 5.9, and 7.9% at 56 DAP, respectively, and 4.9, 7.9, and 11.9% at 70 DAP, respectively. The ST under light shading was higher than that under non-shading at 98 and 112 DAP, although the differences were not significant. The trend in ST was parabolic, with the ST of all treatments increasing from the minimum at 42 DAP to the maximum at 84 DAP, and then decreasing gradually.

Shading significantly affected P/S (Fig. 2e). The P/S under light, moderate, and severe shading decreased by 7.3, 9.0, and 9.8%, respectively, at 56 DAP. Light shading increased P/S by 9.0% at 84 DAP, 10.3% at 98 DAP, and 10.9% at 112 DAP. The P/S of all treatments decreased gradually with time.

### Effects of shading on the vein and leaf thickness

Shading had significant effects on VT (Fig. 2f). The VT under light, moderate, and severe shading was decreased by 5.6, 10.7, and 13.7% at 56 DAP, respectively, and 5.7, 11.6, and 17.0% at 70 DAP, respectively. The VT under light shading was higher than that under non-shading at 98 and 112 DAP, but the differences were not significant. The difference in VT at different collection times was significant. The VT under non-shading reached the maximum at 70 DAP, whereas that of the shading treatment was at 84 DAP.

Shading significantly affected LT (Figs. 1c,f; 2g). The LT under the light treatment was decreased by 7.6% at 56 DAP. The LT under moderate shading was decreased by 10.8% at 56 DAP, 14.2% at 70 DAP, and 9.9% at 84 DAP. Severe shading decreased LT by 9.0% at 42 DAP, 17.6% at 56 DAP, 18.9% at 70 DAP, 13.9% at 84 DAP, 10.2% at 98 DAP, and 9.07% at 112 DAP. There was a significant difference in LT among the different time points. The LT of all treatments increased first and then decreased, whereas the maximum under non-shading and shading was reached at 70 DAP at 84 DAP, respectively.

### Effects of shading on seed histochemistry

**Figure 3:**
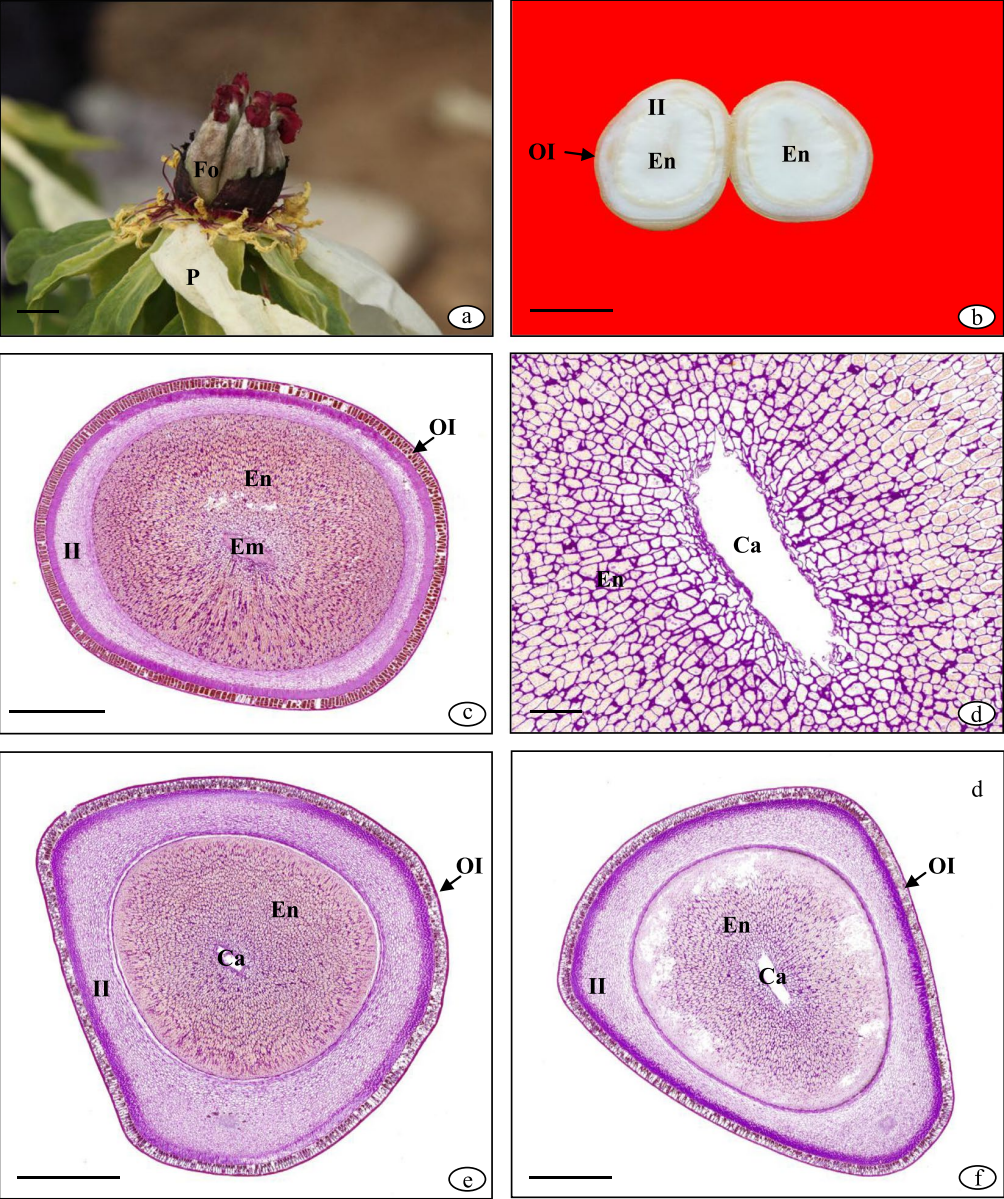
The histochemistry of peony seed. (**a**) Seeds wrapped in follicles after petal shedding; (**b**) Seed vertical section of non-shading at 70 DAP; (**c**) Seed cross section of non-shading at 84 DAP; (**d**) Endosperm cellularization process advanced from outside to inside; (**e**) Seed corss section of non-shading at 70 DAP; (**f**) Seed corss section of severe shading at 70 DAP. Fo: follicles; P: petals; En: endosperm; Em: embryo; OI: outer integument; II: inner integument; Ca: cavity. Scale bars: (**a–b**), 1 cm; (**d**), 200 μm; (**c,e,f**), 1 mm.

Following pollination and petal shedding, the seeds wrapped in follicles gradually increased (Fig. 3a). The tree peony seed is comprised of an outer integument, inner integument, endosperm, and embryo from the outside to inside (Fig. 3b,c). Both the endosperm and embryo experience the free nucleus and cellularization stages. The endosperm is the main tissue for nutrient storage and appeared in a liquid state during the free nucleus stage. The endosperm volume increased as the number of free nuclei increased. The endosperm cellularization process advanced from the outside to inside, and the innermost layer of cells remained nucleus-free until the endosperm was completely cellularized (Fig. 3d). The inner integument gradually degraded and was absorbed by the endosperm cells, and the outer integument also degraded from the inside to outside. Nutrient accumulation in the endosperm proceeded from the outside to inside, with starch accumulating first, followed by gradual increases in protein and fat.

**Figure 4:**
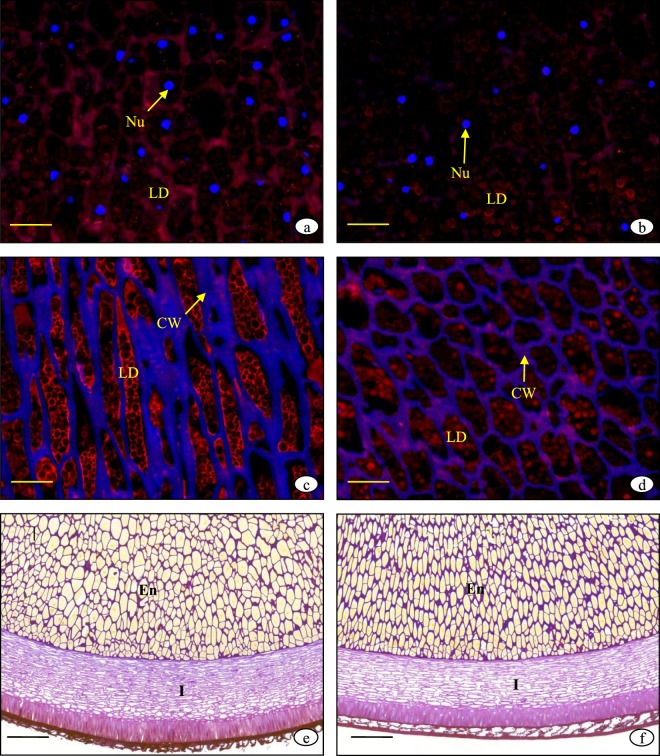
Lipid drops and integument of the peony seed. (**a**) Lipid drops in the endosperm under non-shading at 70 DAP; (**b**) Lipid drops in the endosperm under severe shading at 70 DAP; (**c**) Lipid drops in the endosperm under non-shading at 112 DAP; (**d**) Lipid drops in the endosperm under light shading at 112 DAP; (**e**) Thicker integument under light shading at 112 DAP; (**f**) Thinner integument under non-shading at 112 DAP. Ne: nucleus; LD: lipid drops; CW: cell wall; En: endosperm; I: integument. Scale bars: (**a–d**), 100 μm; (**e,f**), 50 μm.

There was an obvious difference in the seed staining reaction between non-shading and severe shading at 70 DAP. The endosperm cross-section under non-shading was stained dark red with PAS and yellow with naphthol sulfonic acid (Fig. 3e). By contrast, the cross-section under severe shading was stained pink and light yellow (Fig. 3f). This indicated that the endosperm under non-shading contained more starch and protein compared to the one under severe shading. The red spherical lipid drops stained by Nile red in the endosperm section under non-shading were more abundant than that under severe shading (Fig. 4a,b), which indicates that the fat accumulation under non-shading was greater than that under severe shading. At the maturation period, the red spherical lipid drops under light shading were greater in abundance than that under non-shading (Fig. 4c,d). This indicated that there was more fat in the endosperm under light shading than non-shading. The integument under non-shading was significantly thinned and degenerated (Fig. 4f), while that under light shading was still thick (Fig. 4e) and could thus continue to accumulate nutrients.

## Discussion

### The PT under light shading was higher than that under non-shading, which may improve leaf photosynthesis at the maturation period

Plant anatomy links anatomical structures with their functions and their interactions with environment, and allows for the clarification of changes in plant physiology and functions from the microcosmic perspective^13–16^. The leaf is the main organ for assimilation and transpiration and is also the most stress sensitive organ. It is thus of great significance to study the stress resistance of plants by examining leaf anatomical structure^17,18^. Research on leaf anatomical structure has largely focused on the epidermis, mesophyll, and veins^19,20^. Some studies have revealed that leaf structure varies with the environment, with light intensity playing an important role in plant growth and morphological structure^21^.

Light has important effects on photosynthesis, but also acts as an environmental signal in plants for regulating growth, development, physiology, morphological structure, and anatomical structure to adapt to different environments^22–24^. For example, the leaves of *Chrysanthemum morifolium* were thinner under 25% and 15% irradiance and possessed loose palisade tissue and irregularly-arranged spongy mesophyll cells^25^. Adjusting the leaf morphology and anatomical structure allows for the full absorption of light and improvement in photosynthetic rate, thereby promoting growth and development, and ultimately impacting reproduction.

In our previous work, seed development was divided into three periods as follows: rapid growth (before 70 DAP), slow growth (71–98 DAP), and maturation (99–112 DAP)^12^. In this research, LT, VT, PT, and ST all decreased with the decrease in light at the rapid growth stage, which was consistent with the results of *Camellia*^3^ and *Lycoris radiata* var. *radiata*^23^. The thin leaf improved the light capture ability and utilization, as well as the photosynthetic efficiency^26^. The LT and PT under shading were higher than that under non-shading at the maturation stage (Fig. 1e,f). At this stage, the plant appeared to obviously senesce under non-shading, exhibiting leaf yellowing and drying, loss of mesophyll cell water, and decreased cell volume, while the leaf under light shading remained green and in good condition. Similar results were reported in *Brassica napus* L.^27^. In addition, the palisade tissue cells, as the main area for photosynthesis, are directly related to photosynthetic capacity^28^. The fact that PT under light shading was higher than that under non-shading provided an anatomical basis for the improved photosynthetic ability under light shading than under non-shading.

### Light shading delayed integument cell degradation and prolonged seed development and nutrient accumulation

Histochemistry uses specific chemical reagents on plant materials in order observe and determine the substance composition or distribution under the microscope. It combines cell morphology, chemical composition, and function^29^. The endosperm of the tree peony seed is the main tissue for nutrient storage and oil production. Endosperm development begins with a fertilized primary endosperm nucleus. ‘Feng Dan’ has a nuclear endosperm and experiences a free nucleus and cellularization stage, which is consistent with the observation of Dong^30^. The cellularization process of the endosperm proceeded from the outside to inside, and consequently, the starch, protein, and fat also accumulated from the outside to inside.

Dynamic changes in seed storage product accumulation, such as soluble sugar, starch, soluble protein, crude oil, and unsaturated fatty acids, were observed in our previous research^12^. We determined the starch, protein, and crude fat contents of the same samples used for histochemistry analysis, and the results were consistent with our previous observations. In our previous study, we found that nutrient of shading was less than that of non-shading at the rapid growth stage, whereas the fat content under light shading was higher than that under non-shading at the maturation stage^12^. We observed the same result in the stained endosperm section.

At the free nucleus stage, the integument was about half the size of the seed, following which the inner integument degraded as the free nuclei number and endosperm increased. At the cellularization stage, the integument was about a third of the size of the seed, and the inner and outer integument further degraded until the outer integument became hardened and the inner integument shrunk into a thin layer of dense cells. When the seed was mature, the main activity of the endosperm was nutrient conversion rather than nutrient accumulation. The integument degeneration under shading was delayed and seed development was extended, which prolonged nutrient accumulation in the endosperm.

## Conclusions

Our study shows that shading had significant effects on the leaf anatomical structure of oilseed tree peony. At the rapid growth stage (before 70 DAP), the LT under shading was lower than that under non-shading, which reduced light loss and increased the photosynthetic rate. At the maturation stage (99–112 DAP), the LT and PT under light shading were greater than under non-shading, which indicated that leaf senescence was delayed and photosynthetic capacity was increased. At the rapid growth stage, the accumulation of starch and protein under shading was less than that under non-shading, while the fat accumulation under light shading was greater than that under non-shading at the maturation stage. Shading delayed integument degeneration and prolonged seed development and nutrient accumulation. It was thus concluded that leaf structure and seed histochemistry analyses provided structural insights into the improved yield and quality of tree peony seed under light shading conditions.
